# Multiple N-of-1 trials to investigate hypoxia therapy in Parkinson’s disease: study rationale and protocol

**DOI:** 10.1186/s12883-022-02770-7

**Published:** 2022-07-14

**Authors:** Jules M. Janssen Daalen, Marjan J. Meinders, Federica Giardina, Kit C. B. Roes, Bas C. Stunnenberg, Soania Mathur, Philip N. Ainslie, Dick H. J. Thijssen, Bastiaan R. Bloem

**Affiliations:** 1 Center of Expertise for Parkinson & Movement Disorders; Nijmegen, the Netherlands, Department of Neurology, Donders Institute for Brain, Cognition and Behavior, Radboud University Medical Center, Nijmegen, The Netherlands; 2 IQ Healthcare, Radboud Institute for Health Sciences, Radboud University Medical Center, Nijmegen, The Netherlands; 3 Department of Health Evidence, Radboud Institute for Health Sciences, Radboud University Medical Center, Section Biostatistics, Nijmegen, The Netherlands; 4 Department of Neurology, Donders Institute for Brain, Cognition and Behavior, Radboud University Medical Center, Nijmegen, The Netherlands; 5 Department of Neurology, Rijnstate Hospital, Arnhem, Netherlands; 6 UnshakeableMD, Oshawa, ON Canada; 7 Center for Heart, Lung and Vascular Health, School of Health and Exercise Sciences, University of British Columbia, Kelowna, Canada; 8 Department of Physiology, Radboud University Medical Center, Nijmegen, The Netherlands

**Keywords:** Parkinson’s disease, Hypoxia, Treatment, Disease-modifying, Mitochondrial dysfunction, Clinical trial

## Abstract

**Background:**

Parkinson’s disease (PD) is a neurodegenerative disease, for which no disease-modifying therapies exist. Preclinical and clinical evidence suggest that hypoxia-based therapy might have short- and long-term benefits in PD. We present the contours of the first study to assess the safety, feasibility and physiological and symptomatic impact of hypoxia-based therapy in individuals with PD.

**Methods/Design:**

In 20 individuals with PD, we will investigate the safety, tolerability and short-term symptomatic efficacy of continuous and intermittent hypoxia using individual, double-blind, randomized placebo-controlled N-of-1 trials. This design allows for dose finding and for including more individualized outcomes, as each individual serves as its own control. A wide range of exploratory outcomes is deployed, including the Movement Disorders Society Unified Parkinson’s Disease Rating scale (MDS-UPDRS) part III, Timed Up & Go Test, Mini Balance Evaluation Systems (MiniBES) test and wrist accelerometry. Also, self-reported impression of overall symptoms, motor and non-motor symptoms and urge to take dopaminergic medication will be assessed on a 10-point Likert scale. As part of a hypothesis-generating part of the study, we also deploy several exploratory outcomes to probe possible underlying mechanisms of action, including cortisol, erythropoietin and platelet-derived growth factor β. Efficacy will be assessed primarily by a Bayesian analysis.

**Discussion:**

This evaluation of hypoxia therapy could provide insight in novel pathways that may be pursued for PD treatment. This trial also serves as a proof of concept for deploying an N-of-1 design and for including individualized outcomes in PD research, as a basis for personalized treatment approaches.

**Trial registration:**

ClinicalTrials.gov Identifier: NCT05214287 (registered January 28, 2022).

**Supplementary Information:**

The online version contains supplementary material available at 10.1186/s12883-022-02770-7.

## Background

Parkinson’s disease (PD) currently affects 10 million people worldwide and its prevalence is projected to rise exponentially in the coming decades [1]. Several symptomatic treatments are available, the mainstay of which has been levodopa for over half a century. Many patients continue to experience significant disability, despite deployment of all available management strategies. Therefore, additional treatment modalities are needed.

Anecdotal evidence from individuals with PD suggests that ascending to high-altitude areas occasionally improves motor symptoms of PD, in a subacute way. These findings were recently confirmed in a survey that we conducted among individuals with PD who had recently been on vacation (Janssen Daalen et al., manuscript submitted). We hypothesize that the positive effect of altitude on PD symptoms results from the decreased arterial oxygen tension at high altitude, which serves as an acute bodily stimulus for multisystem adaptations that potentially have protective effects on cellular homeostasis and survival [2, 3]. Therefore, altitude simulation has been the topic of research for potential therapeutic application in a variety of diseases.

Preclinical studies have suggested that hypoxia provokes release of survival-enhancing neurotransmitters. Specifically, the short-term clinical effects of hypoxia therapy appear to be related to augmented dopamine release from the substantia nigra [4–9]. Hypoxia therapy may improve parkinsonian symptoms via stabilization of hypoxia inducible factor 1 (HIF-1) and its downstream pathway, which in turn activates tyrosine hydroxylase (TH), the main rate-limiting enzyme in the production of dopamine [10, 11]. Several studies have demonstrated that HIF-1 stabilization leads to an increase in TH production, and consequently a rise in cellular dopamine content [8–13]. In addition, hypoxia protocols have a different influence on sympathetic nervous system activity, which regulates the body’s stress response [12, 14–16]. For example, long-term hypoxia increases noradrenalin-adrenalin ratio [12], which might ameliorate non-dopaminergic symptoms [17]. Taken together, these converging observations in animals and humans, provide a rationale that explains the potential positive effects of hypoxia on PD symptoms.

In addition to the short-term effects mentioned above, other studies also suggest that repeated exposure to hypoxia induces an evolutionary well-conserved adaptive mechanism. This adaptive response involves improves cellular energy metabolism as impaired by mitochondrial dysfunction, inhibits oxidative stress and induces adaptive plasticity, suggesting that in addition to the acute symptomatic effects, hypoxia might also exert long-term neuroprotective effects [18–20]. The concept behind these neuroprotective effects is the phenomenon of hypoxic preconditioning (HPC): induction of a sub-toxic hypoxic stimulus to improve the (systemic) tolerance of cells and tissues to subsequent more severe toxic stimuli. Although there is debate regarding the most potent hypoxia treatment regimen, clinical and preclinical evidence suggests that these effects are more pronounced when applied using a regime of intermittent hypoxia therapy (IHT) as compared to continuous hypoxia, meaning that hypoxia is present for short periods (i.e., minutes), interspersed with short periods of normoxic recovery. To date, hypoxia therapy, mostly IHT, has been used in a variety of populations, including fragile ones such as individuals with spinal cord injury, COPD, cardiac morbidity and multimorbidity, without any significant side effects [21–30]. However, the safety, feasibility and efficacy of hypoxia-based therapy have not been systematically investigated yet in individuals with PD. In this exploratory trial, we will assess the potential of hypoxia-based therapy in PD by assessing the physiological response to hypoxia, while also measuring the short-term symptomatic effects. To assess both continuous and intermittent hypoxia treatment regimens, this trial will deploy a double-blind, randomized placebo-controlled N-of-1 design, which allows for testing all selected hypoxia protocols in all participants [31].

### Study objectives

#### Primary objectives

(i) to evaluate the safety and feasibility of both intermittent and continuous hypoxia therapy in individuals with PD under well-controlled circumstances.

(ii) to explore the responsiveness of acute symptomatic outcome measures of intermittent and continuous hypoxia therapy in individuals with PD under well-controlled circumstances.

#### Secondary objectives

(iii) to assess the acute symptomatic effects on selected subjective and standardized symptom scales.

(iv) as a hypothesis-generating addition, to explore the potential mechanisms of (intermittent) hypoxia therapy on PD.

### Hypothesis

We hypothesize that short-term hypoxia-based protocols are safe to apply in individuals with PD without cardiorespiratory comorbidity, and that IHT has short-term effects on dopamine, noradrenalin and stress-responsive symptoms in PD.

## Methods/Design

### Multiple N-of-1 trials

In this study, we will conduct multiple series of randomized, double-blind and placebo-controlled N-of-1 trials (also known as single participant cross-over trials). This design includes the testing of multiple hypoxia protocols in every participant, and it allows for the analysis of treatment effects in the individual participant (in addition to group effects) because the participant serves as his or her own control. Lastly, this design can result in a higher power when considering small populations as compared to other traditional designs, making N-of-1 trials appropriate for studying rare diseases and personalized treatment [32, 33]. N-of-1 trials are especially suitable to investigate treatments in chronic, symptomatic conditions, where period effects (i.e. changes in disease state) and carry-over effects (i.e. lingering treatment effects) are limited. Given the slowly progressive nature of PD with relative stable symptoms, several N-of-1 trials have already been successfully performed to study symptomatic treatments in PD [34, 35].

### Study population

Twenty individuals with a diagnosis of PD (established by a neurologist according to the international Movement Disorder Society criteria) and Hoehn & Yahr stages between 1.5 and 3 will be included. Higher Hoehn & Yahr stages will be excluded for two reasons: firstly because of the significantly greater damage to dopaminergic pathways in advanced PD, which might limit the identification of nigrostriatal-mediated effects of hypoxia-based interventions; and secondly because of the high burden of testing participants in the OFF state in this more severely affected population. We aim to enrich our study population with individuals that have experienced (subjective) positive symptomatic effects at high altitude. In addition, we aim to include at least five individuals without prior experience with the positive effects of high altitude on their symptoms. This enrichment approach ensures that participants are more likely to be responders and allows us to validate our approach to identify each individual’s optimal protocol for clinical benefit. We believe that the selection bias introduced by our approach is justifiable because of the study’s explorative nature. In addition, PD is a very heterogeneous disease, for which a one-size-fits-all treatment approach is unlikely to be successful. Therefore, we wish to investigate for which individuals this therapy could be beneficial and the proposed approach is likely to be most promising. Main exclusion criteria relate to cardiorespiratory comorbidity and unstable PD medication. All inclusion and exclusion criteria can be found in Table 1.

**Table 1:** Inclusion and exclusion criteria

Inclusion criteria	Exclusion criteria
Clinical diagnosis of Parkinson’s disease by a movement-disorder specialized neurologist	Individuals with diseases leading to restrictive and obstructive pulmonary diseases, pulmonary diffusion deficits, apnea and cardiac output deficits, such as pulmonary fibrosis, COPD, sleep apnea or excessive alcoholic intake, and congestive heart failure respectively
Hoehn and Yahr staging 1.5 to 3	Arterial blood gas abnormalities at screening day
Age > 18 years	Individuals with shortness of breath or other airway or breathing-related inconvenience related to lack of dopaminergic medication will be excluded
Participant can provide informed consent	Inability to comply to intervention in off-medication condition (for example due to extreme discomfort, distress or severe head tremor due to being OFF, i.e. without dopaminergic medication)
	Individuals with unstable dopaminergic medication dose (changes in the last month)
	Individuals likely to start dopaminergic treatment in the next month, also judged by their treating neurologist
	Individuals with active deep brain stimulation
	Individuals unable to provide informed consent

### Sample size

Because this is the first study in which the clinical effects of hypoxia therapy will be measured in PD and because of the study’s exploratory nature, a formal sample size calculation cannot be performed. A previously published power and sample size simulation study aggregated N-of-1 trials with multiple cycles of intervention and placebo per participant, and found that under certain assumptions N-of-1 trials needed only one-third of the sample size of an RCT to reach a similar power and type I error [36]. This was confirmed in a recent aggregated N-of-1 trial that investigated the effectiveness of the drug mexiletine in myotonia, in which the aggregated N-of-1 trial showed comparable treatments effects on a personalized Likert scale (i.e., the same outcome measure as we propose) with inclusion of 11 participants vs 57 participants in a traditional cross-over RCT [32]. Our proposed sample size of 20 was motivated by this work, in combination with the sample size consensus for feasibility studies of 20–30 [37].

The observed effect sizes and standard deviations of the different hypoxia protocols that are found to be clinically meaningful at the participant group level in this study (*N* = 20) will be used to simulate a power and sample size calculation for a future follow-up study that will be powered for efficacy using the data generated in this study.

### Recruitment, screening and inclusion

Individuals with PD will be recruited via the web-based ParkinsonNEXT recruitment website (www.parkinsonnext.nl) and, if necessary, from the outpatient registry of our university medical center – all patients on this list have already consented to be contacted for research purposes. After a first telephone contact with the coordinating investigator, potential participants will receive an email with detailed information about the study, including an overview of necessary time investment and the risks of participation, as well as the informed consent form. Specifically, we will ask participants with a positive altitude experience to share this project in their own networks of hikers or mountaineers.

Individuals will have a consultation by telephone conducted by the coordinating investigator to answer questions about the research and the informed consent. If the participant is still interested, screening questionnaires will be sent before the physical screening day. The coordinating investigator will contact the participant by phone to check if potential exclusion criteria are met, to prevent any unnecessary participant visits. This process will be registered in a pre-screening list. In this way, a transparent overview of study pre-selection will be available. The informed consent will be completed before initiation of the screening day. The participant has the right to withdraw consent at any moment during the study period. Drop-outs will be replaced.

### Intervention

We will study the response to multiple exposures of different pre-selected hypoxia protocols per participant, as at present there is no conclusive evidence for the effect of different hypoxia stimuli. This will optimize the chances for any changes in outcomes being identified, and allowing the possibility of defining the optimal dose of hypoxia as a therapeutic intervention individually. The selection of interventions was based on multiple studies that investigated and reviewed the hypoxia regimes that are currently perceived as being most effective [38–43]. In this N-of-1 trial design, every participant will receive two sets of five different conditions, with one being the placebo and the other four variants of an active intervention, consisting of either intermittent or continuous hypoxia at FiO2 0.127 (~ 4000 m) and 0.163 (~ 2000 m). All interventions are administered twice to enhance intra-individual discriminative power towards a sufficiently low or high probability (see *Statistical Analysis*). As it is shown that hypoxic preconditioning effects may linger for up to four days [44], a wash-out of at least five days between sessions will be built-in. Once a week, one of the following five conditions will be administered following the order resulting from a Latin square randomization (see *Statistical Analysis*):

Active interventions:

- Continuous hypoxia for 45 min, at ~ 2000 m (16.3% O_2_)
- Continuous hypoxia for 45 min, at ~ 4000 m (12.7% O_2_)
- Intermittent hypoxia with 5 × 5-min at ~ 2000 m (16.3% O_2_), interspersed with 5-min normoxic recovery
- Intermittent hypoxia with 5 × 5-min at ~ 4000 m (12.7% O_2_), interspersed with 5-min normoxic recovery
  Placebo intervention:
- Continuous normoxia for 45 min (20.9% O_2_)

The intervention will be performed at Radboud university medical center in Nijmegen, the Netherlands. A commercially available hypoxicator will be used to deliver the hypoxic bout (*B-cat High Altitude*, the Netherlands). The hypoxicator (as an oxygen concentrator) is ISO 13485:2016 certified and uses a process called *pressure swing adsorption* to filter ambient air and to extract oxygen from that air. The principle is widely used in oxygen concentrators, but is inversely applied in the case of hypoxicators. Instead of oxygen-rich air, the resultant hypoxic gas is administered to the patient [45]. The hypoxicator is connected via medical-grade tubes to two large (> 50L) reservoir air bags that buffer the hypoxic gas mixture, which optimizes stability of the delivered FiO2. Intermittent hypoxia and the placebo situation is administered by switching a three-way Hans Rudolph® valve to either room air or the hypoxic circuitry. The intraday and between-day reliability of the hypoxicator were tested on multiple days before application in participants. Intraday variability was low (average range 0.2 percent points around desired FiO2) and between-day variability was within 0.1 percent points of desired FiO2. Seven days before the start of the first intervention, a one week at-home registration of self-reported outcomes of symptom severity will be conducted by a short daily morning survey. This assures a solid baseline of symptom severity that will be used in the Bayesian analysis (see *Statistical Analysis*).

### Study procedures

#### Screening

Before inclusion, potential participants will come to the hospital for a dedicated screening day, during which extensive safety screening will be conducted. Firstly, this will consist of standard pulmonary function testing (PFT) using spirometry to assess the presence of any previously unknown restrictive or obstructive pulmonary diseases. Second, a carbon monoxide diffusion test for any unknown diffusion deficits is conducted to exclude any unknown comorbidity that might pose a health risk during the study. Finally, peak cough flow and mean inspiratory pressure (MIP) are assessed, as well as subjective respiratory problems via a selection of questions that predicted PD-related respiratory problems (Supplementary Table 1) in a recent study [Van de Wetering et al., submitted]. All participants will also undergo an electrocardiogram (ECG) to screen for any cardiac abnormalities such as dysrhythmias or signs of ischemia.

Subsequently, participants will be blindly exposed to stepwise decreasing levels of FiO2 until an FiO2 of 0.127 is administered fully. This will be performed in four steps. Before every step (step duration of approximately 5 min), arterial blood gas (ABG) and vital parameters are evaluated and compared to the pre-determined stop criteria (Supplementary Table 2). Vital parameters including peripheral oxygen saturation will be collected every 5 min and will be correlated to the ABG results to evaluate whether these vital parameters reflect the ABG results. If one or more of the stop criteria are met and the breathed-in air does not yet contain an FiO2 of 0.127, the intervention will be stopped. Moreover, the participant will then be excluded from further interventions and will be replaced.

#### Pilot phase

To ensure optimal safety, a pilot study of the first two participants selected for this study protocol will be deployed before conducting this study protocol at full pace. During the screening procedure, all individuals will be exposed to gradually decreasing FiO2’s until an FiO2 of 0.127 (~ 4000 m altitude) is reached. In these two individuals, the most intense intervention (continuous hypoxia at FiO2 0.127) will be administered for 45 min, to evaluate whether exhaustion in individuals with PD will limit the maximum safe duration as clinical experience is limited. In the remaining participants, the 4000 m intervention will be administered until ABG parameters have stabilized. When no serious adverse events occur, the protocol will be continued. During this pilot phase, the same stop criteria will be adopted as during the interventions.

#### Intervention

After completion of the screening day, participants will visit the hospital on a weekly basis, for 10 consecutive weeks. Treatments and in-hospital assessments will be conducted in the practically-defined OFF phase and will therefore commence in the morning. The treatment session in the OFF state is preceded by a baseline clinical assessment and is immediately followed by a post-intervention clinical assessment and an assessment after 30 min (Table 3). The intervention is administered in a hospital by an experienced lab technician, who also continuously monitors participants during and after the intervention. During the intervention, the inhaled FiO2 is measured continuously using the COSMED® metabolic system (*Quark CPET* metabolic cart for cardiopulmonary testing, COSMED Srl, The Metabolic Company, Italy), which also measures peripheral oxygen saturation, blood pressure, respiratory rate and tidal volume. In addition, the subjective participant experience regarding dizziness, discomfort and stress is registered by the lab technician during the intervention. In addition, to maximize safety of the participants during the intervention, clear stopping criteria are defined. If these are met, the lab technician will halt the intervention (‘Stopping Criteria’, Supplementary Materials).

### Randomization and blinding

#### Randomization

**Fig. 1:**
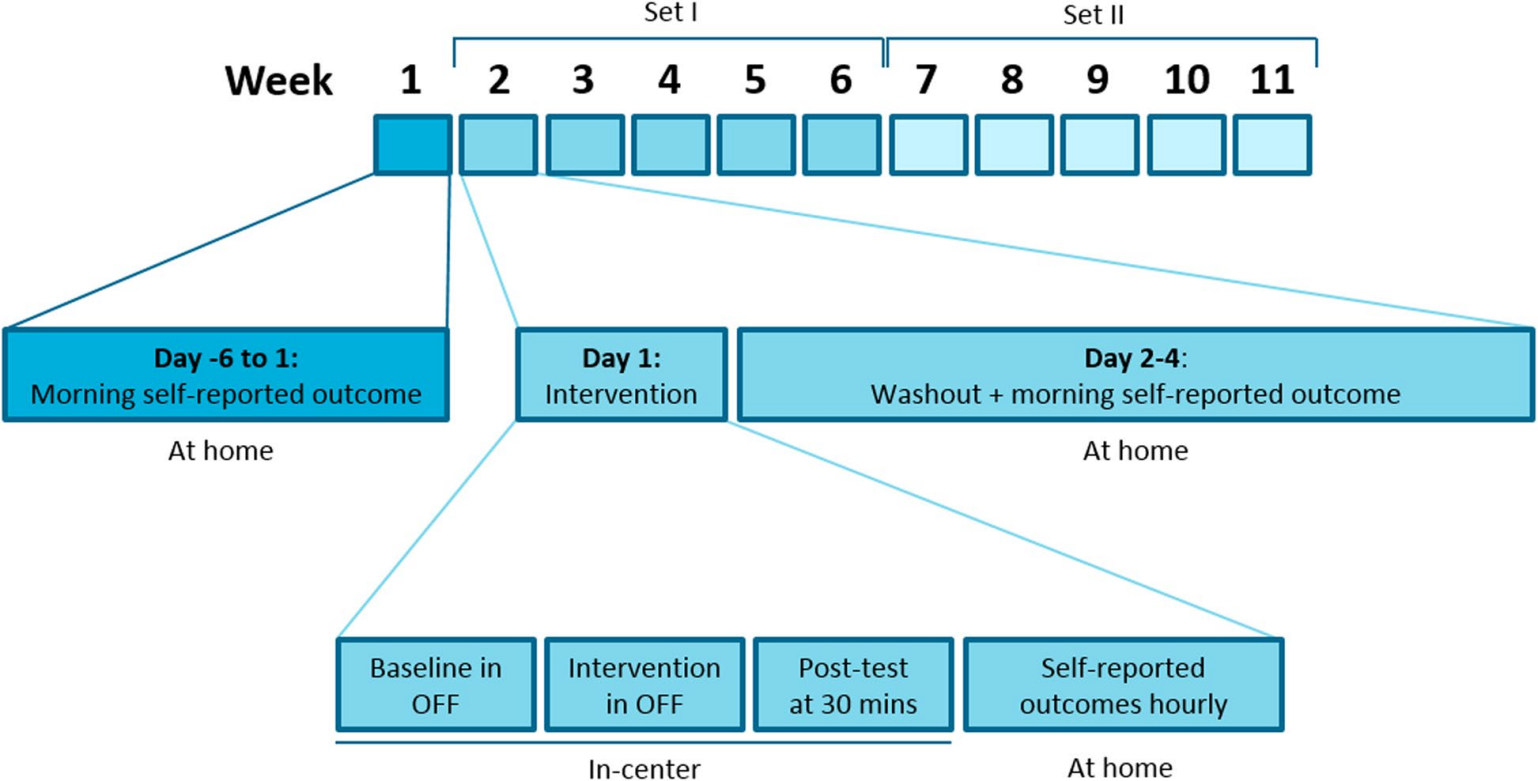
Design of the self-reported outcomes scoring in the multiple N-of-1 trials of every individual patient

Subjects will be equally divided in 5 groups with different interventions order according to a Latin square design (5 periods/interventions). Allocation to intervention order will be randomized by a computer-generated randomization scheme. Interventions will be randomized at study start in two sets. As depicted in Fig. 1, set I will be randomized for the first five interventions and set II for the second five interventions. This reduces the risk of placebo effects in Set II potentially provoked by any positive effects that may have been experienced in Set I. This balanced randomization scheme minimizes bias introduces by the resulting order effect and period effect. Carry-over effects are minimized by ascertaining a sufficient wash-out period between every treatment.

#### Blinding

The intervention is double-blind for the participant and assessor, but not for the lab technician (because of safety and monitoring purposes). Ventilation during the administration is expected to increase by ~ 20%, mostly due to increased tidal volume and less often due to respiratory rate [46]. Risk of unblinding due to these limited ventilatory effects is unlikely and has not been reported in previous hypoxic experiments [28, 46–48]. Additionally, complaints of discomfort or shortness of breath are sometimes reported, regardless of the administered FiO2, indicating that this will not hamper blinding [23–25]. Changes in respiratory parameters will be available for the lab technician during the experiments, but will not be disclosed to the assessor. The assessor will only enter the lab after the intervention has been fully completed and will not have access to the vital parameters and blood parameters of the participant from which it might be possible to infer the assigned intervention. This problem is also mitigated by adding the placebo condition. We will assess the success of the blinding procedure by asking the participant in what sequence the different treatments were administered after the first and second treatment set.

### Outcomes and measurements

#### Primary outcomes

The main primary outcomes of this study are the safety and feasibility of IHT under well-controlled circumstances in a lab-based setting. Safety outcomes will be measured by the lab technician during the intervention and by the reporting of adverse events. The feasibility questionnaire (Supplementary Table 3) is designed together with a patient researcher. The questionnaire’s content is based on the main categories from a widely used feasibility framework [49]. Statements in every category (e.g. acceptability, expectancy) were subsequently inspired by previously published healthcare feasibility questionnaires. Primary outcomes are summarized in Table 2.

**Table 2:** Primary outcomes

**Primary outcome variable**	*Measurement frequency*
Nature and number of adverse events	Every 10 min during intervention, up to one hour post-intervention, one time next morning post-intervention
Self-reported dizziness, discomfort and stress on a ten-point scale	Every 10 min up to one hour post-intervention, one time next morning post-intervention
Number of serious or irreversible adverse events	Every 10 min, up to one hour post-intervention, one time next morning post-intervention
	*Measured continuously for safety, recorded:*
- Blood pressure systolic-diastolic	Baseline and every 5 min—> 30 min post-intervention
- Heartrate	Baseline and every 5 min—> 30 min post-intervention
- Respiratory rate	Baseline and every 5 min—> 30 min post-intervention
- Oxygen saturation	Baseline and every 5 min—> 30 min post-intervention
Feasibility questionnaire (total score and subscores) (in *Supplementary Materials*)	After 1^st^, 5^th^ and 10^th^ intervention

### Secondary outcomes

#### Self-reported outcomes

In this exploratory study, we will use a battery of outcomes to determine which symptoms or signs might respond to hypoxia. In accordance with accepted N-of-1 trial design recommendations and previously conducted trials [32, 50, 51], we have chosen to predefine personalized (self-reported) outcomes for each individual, scored on a 10-point Likert scale. Contrary to standardized PD scales, these outcomes better reflect important effects for the individual, and are potentially more sensitive to subtle treatment effects, reducing the risk of type II errors. Therefore, we included three participant-rated outcomes that assess on a 10-point Likert scale (allowing half points) general symptom impression, the urge to take a next dose of dopaminergic medication and the effect on one specific symptom that participants can choose themselves, based on goal attainment scale principles (Table 3). This symptom must fulfill the following criteria: it must fluctuate in severity throughout the day or between days, changes in its severity should be swiftly apparent and, if applicable, it is a symptom that previously improved at high altitude. To ensure a baseline score on these outcomes, participants will report these on a daily basis in the morning (in OFF) during the baseline period, i.e. 7 days preceding the start of the intervention period. On intervention days, participants will report these self-reported outcomes pre-intervention, directly post-intervention, 30 min post-intervention and from that moment on, another five times, on an hourly basis. In addition, these will be measured once every morning (i.e. in OFF) for the next three mornings after the intervention.

**Table 3:** Secondary outcomes

Self-reported outcome variable	Researcher	Participant	Moment of measurement (in addition to baseline)	Measurement unit
**General symptom impression**		X	Directly after intervention and post-intervention 30 min, 1 h, 2 h, 3 h, 4 h and 5 h, and once every morning on the next three days	10-point Likert scale allowing half points
**Urge for dopaminergic medication** on the usual moments of intake		X	Directly after intervention and post-intervention 30 min, 1 h, 2 h, 3 h, 4 h and 5 h, and once every morning on the next three days	10-point Likert scale allowing half points
**Participant-selected symptom** (goal attainment scale)		X	Directly after intervention and post-intervention 30 min, 1 h, 2 h, 3 h, 4 h and 5 h, and once every morning on the next three days	10-point Likert scale allowing half points
**Motor symptom severity** (self-selected, symptom that improved most during high altitude exposure, if applicable)		X	Directly after intervention and post-intervention 30 min, 1 h, 2 h, 3 h, 4 h and 5 h, and once every morning on the next three days	10-point Likert scale allowing half points
**Motor symptoms** MDS-UPDRS part III	X		30 min post-intervention	Total score and subscores
**Hand tremor** Accelerometry/gyroscope	X		Directly post-intervention, 30 min post-intervention	Amplitude
**Rapid alternating movements** Accelerometry/gyroscope	X		30 min post-intervention	Amplitude
**Bradykinesia** Modified Perdue pegboard test	X		30 min post-intervention	Number of pins
**Gait** Timed Up & Go Test	X		30 min post-intervention	Steps and seconds
**Balance** MiniBESTest	X		30 min post-intervention	Total score and subscores
**Non-motor symptoms** MDS Non-motor Scale symptoms	X		30 min post-intervention	10-point Likert scale (half points)
**Stress** Heartrate variability	X		During intervention, directly after intervention, 30 min post-intervention	RR interval

The measurement scheme of all outcomes is displayed in Supplementary Fig. 1. Patients will report all self-reported outcomes digitally.

#### Assessor-rated outcomes

Assessor-rated outcomes are summarized in Table 3. These consist of gold-standard general motor tests (Movement Disorders Society Universal Parkinson’s Disease Rating Scale part III, or MDS-UPDRS part III) and non-motor tests (symptoms from the MDS Non-motor symptoms scale, or NMSS), supplemented with specific tests for bradykinesia (Purdue pegboard test [52]), gait (Timed Up & Go Test [53]), balance (Mini-BESTest [54]) and quantified versions of the UPDRS items on tremor and pronation-supination (performed using accelerometry). These secondary outcomes are measured 30 min post-intervention, after which hypoxic preconditioning is believed to have its initial first window effects peak. After 30 min, the acute effects of hypoxia therapy peak, as shown in people with a cervical spinal cord injury [55], with the second window only occurring approximately 3 h post-intervention.

Various selected outcomes, including the UPDRS part III, are sensitive to the Hawthorne effect: individuals with PD try to perform as good as possible due to the awareness of being observed. To mitigate such effects, the placebo condition is added, for which the multiple N-of-1 design is also particularly suited [31, 36, 56].

Baseline characteristics that will be collected include age, gender, H&Y, quality of life (Parkinson’s disease questionnaire 39 [57]) and other medication. Potential effect modifiers include levodopa-equivalent dose (LED), sleep quality (4-point ordinal scale) and physical activity (International Physical Activity Questionnaire – Short Form, IPAQ-SF [58]) and these will be measured during every pre-intervention phase.

#### Mechanistic markers

Lastly, three markers will be measured in serum, which serve as a hypothesis-generating addition to our study. These measures may provide insight into potential pathways involved in the (individual) responses to hypoxia in our study. These measures include platelet-derived growth factor receptor β (PDGFRβ), cortisol and erythropoietin (EPO).

PDGFRβ is a pericyte-shedded marker in response to hypoxia [59–62] and is associated with blood–brain barrier (BBB) permeability [60, 61, 63–65]. Disruption of the BBB is a central process involved in PD pathophysiology [66] and therefore, acute effects of hypoxia therapy on PDGFRβ would give insight in the influence that hypoxia might have on BBB integrity in the long term [65].

Cortisol is a marker of physiological and mental stress and is hypothesized to rise during systemic hypoxic challenges [67]. As stress is one of the main determinants of variations in symptom severity, we investigate whether cortisol release might be associated with the short-term symptomatic efficacy of hypoxia-based interventions. This will give insight in whether hypoxia has beneficial influence on the stress system, or that the physiological stress results in fatigue and mental stress, thus worsening PD symptoms in the short-term. Cortisol is measured twice pre-intervention and three times post-intervention, because of its concentration changing with circadian rhythm.

EPO is a protein that is primarily shedded peripherally by the kidney. Although it is employed as a marker of hypoxic dose, attention for its role in neuroprotection has risen in recent years [68]. In preclinical PD models, it prevents neurotoxicity and preserves neuronal functioning [69, 70].

All outcome measures are summarized in further detail in Supplementary Fig. 1.

### Statistical analysis

All data will be collected using direct entry in CastorEDC, a widely used electronic data capture system for clinical data.

#### Analysis of primary study parameters

Safety outcomes will be analyzed using descriptive statistics of total number and percentages of adverse events, number of serious reversible or irreversible adverse events and vital parameters. Feasibility outcomes will be analyzed using descriptive statistics of feasibility outcomes including the feasibility questionnaire sum score and domain-specific scores.

### Secondary study parameters

#### Bayesian analysis

We will use a Bayesian model for the analysis of individual and aggregated N-of-1 trial results. This model allows for a direct estimation of the posterior probability that a treatment results in a clinically beneficial effect [32, 71–73]. The treatment effects resulting from the four different hypoxia protocols will be estimated per level of interest (i.e. the individual and group level). Every secondary outcome scored using the 10-point Likert scale will be modeled assuming a normal distribution centered around the patient’s true mean and variance for each protocol. If the posterior probability of reaching a 0.75-point difference between the secondary outcome after treatment and at baseline is greater than 80%, treatments are considered effective. Treatments are considered ineffective if the same posterior probability is less than 20%. Every treatment day has its own baseline (pre-intervention tests are performed) in order to further reduce period effects. The minimal clinically important difference for the secondary outcome MDS-UPDRS part III and Mini-BESTest is 4. One additional analysis will be performed including baseline characteristics and effect modifiers (see *Assessor-rated outcomes)* as covariates. Bayesian analyses will be performed using *JAGS* version 3.4.1 [74], run from *R* [75] using the package *rjags* [76].

#### Frequentist analysis

For comparison with the Bayesian analysis we will also perform an explorative analysis of the secondary outcomes. For those that are normally distributed, we will use dependent *t* tests to calculate mean treatment effects, significance levels, and confidence intervals on group level. *P* values are 2-sided, and *P* < 0.05 will be the threshold for significance for all tests. Analyses will be performed using *R* [75]*.*

### Monitoring and registration

#### Quality assurance and monitoring

The study will be monitored regarding the health, safety and rights of participants, protocol adherence and quality of data and data reporting during this trial at study initiation, twice during the study (on-site) and once at study completion. On-site monitoring visits (performed by Radboudumc or designee) will assess the progress of the study, study procedures, used study materials and identify any concerns that result from review of the subject Informed Consent documentation, study records, collected data and study management documents. The study monitor will also ensure the Investigator adheres to all applicable regulations.

#### Data safety monitoring board

A data safety monitoring board (DSMB) will be established, which will consist of a PD-specialized neurologist, an anesthesiologist, a pulmonologist, a biostatistician and a patient representative. A first interim analysis of the safety outcomes will be performed after the pilot phase of the first two subjects that are halfway through the treatment protocol (which means every treatment is already administered once), to provide the DSMB with the latest data on adverse events and recruitment. The second interim analysis after 7 participants have completed their protocol will give insight in primary as well as secondary outcomes.

## Discussion

In this study, we propose multiple N-of-1 trials to investigate the merits of hypoxia-based interventions as a new symptomatic therapy in persons with PD. For the first time, this design offers a unique opportunity to test for the first time the safety, feasibility and short-term efficacy of various interventions in this unexplored therapeutic area in PD. Therefore, a wide variety of participant-reported and assessor-rated outcome measures will be deployed.

Hypoxia-based therapy has been applied extensively in research in a wide spectrum of healthy participants and individuals with medical conditions, and both short- and long-term effects have been investigated. Examples of previously studied treatment goals in various populations include rehabilitation in spinal cord injury (SCI) [55, 77–81], cardiorespiratory control in type I and II diabetes [82, 83], endurance and exercise tolerance and performance in healthy and geriatric individuals [24, 25, 27, 29, 84, 85], cognitive performance in geriatric and elderly individuals [22, 26, 28, 86], cardiovascular risk factors in obese individuals [87], reducing acute mountain sickness [88], and training of respiratory dysfunction [42, 48, 89–91]. However, clinical parameters or symptomatic efficacy of hypoxia-based therapy have thus far never been studied in PD, even though the aforementioned underlying working mechanisms of hypoxia would make PD an attractive disorder to study. One earlier brief report investigated the effects of (unspecified) IHT on the hypoxic ventilatory response in PD and found markedly reduced hypoxic ventilatory response, indicating a suboptimal response in breathing frequency to hypoxic challenges [48]. Because there is such limited previous experience with delivering hypoxia to persons with PD, several theoretical concerns must be addressed in this study proposed here. First, because respiratory abnormalities can already be observed in relatively early stages of PD [92], the safety and feasibility profile of different hypoxia protocols must be established first; this is one of the goals of the present study. Second, the short-term as well as long-term effects should be investigated separately, as different mechanisms might be involved [Janssen Daalen et al., manuscript in preparation]. With regard to short-term effects, we might counter both beneficial and harmful effects (such as stress, increased oxidative stress), and the hypoxic dose for which this balance is optimal remains to be established in PD.

This exploratory study will provide the first insights into the potential of hypoxia-based therapy in PD. Additionally, our study might yield hypothesis-generating insights regarding its underlying working mechanisms. At the same time, the findings might also improve our understanding of the mechanisms of respiratory involvement in PD and on motor and non-motor symptom variability, that can be derived from the weekly administered neurological test battery. The findings in this study might partly be extrapolated to other neurodegenerative diseases, such as Alzheimer’s disease [40] or mitochondrial diseases [20]. Although the mechanisms of hypoxia-based interventions remain to be fully elucidated, we believe this rationale warrants the first well-controlled randomized trial of hypoxia-based interventions in PD.

## Supplementary Information

**Additional file 1:** **Supplementary Table 1. **Orientating questionnaire for respiratory problems in PD [Van de Wetering et al., submitted]. **Supplementary Table 2. **Stop criteria. **Supplementary Table 3. **Feasibility questionnaire, scored on 10-point Likert scale. **Supplementary Figure 1. **Summary of outcome measurements

**Additional file 2:** SPIRIT checklist for interventional trials

## Data Availability

Anonymized data will be shared with The Michael J. Fox Foundation for Parkinson’s Research (the study funder). This data may be kept for storage at a central repository either hosted by The Michael J. Fox Foundation, its collaborators, or consultants and will be kept indefinitely. Anonymized data will be made publicly available by the Foundation for the intended use of research in Parkinson’s disease as well as other biomedical research studies that may not be related to Parkinson’s disease.

## Abbreviations

PD: Parkinson’s disease
HIF-1: Hypoxia-inducible factor 1
TH: Tyrosine hydroxylase
HPC: Hypoxic preconditioning
MiniBES: Mini Balance Evaluation System
MDS-UPDRS: Movement Disorders Society Unified Parkinson’s Disease Rating scale
IHT: Intermittent hypoxia therapy
SCI: Spinal cord injury
DSMB: Data safety monitoring board
PFT: Pulmonary function testing
MIP: Mean inspiratory pressure
ECG: Electrocardiogram
ABG: Arterial blood ga
LED: Levodopa-equivalent dose
IPAQ-SF: International Physical Activity Questionnaire – Short Form
PDGFRβ: Platelet-derived growth factor receptor β
EPO: Erythropoietin
BBB: Blood–brain barrier
MJFF: Michael J. Fox Foundation for Parkinson’s Research
