# Supplementary material for: Multiple N-of-1 trials to investigate hypoxia therapy in Parkinson’s disease: study rationale and protocol
Source: BMC Neurol. 2022 Jul 14;22:262. doi: 10.1186/s12883-022-02770-7 (PMC9281145; doi:10.1186/s12883-022-02770-7)

# Supplementary materials

**Supplementary Table 1:** Orientating questionnaire for respiratory problems in PD [Van de Wetering et al., submitted]

1. **Did you experience in the last year…**

| … problems with breathing? | No | Yes: |  | Seldomly | Occasionally | Often | Continuously |
| --- | --- | --- | --- | --- | --- | --- | --- |
| … the feeling you are short of breath? | No | Yes: |  | Seldomly | Occasionally | Often | Continuously |
| … a tight feeling on your chest complicating easy breathing? | No | Yes: |  | Seldomly | Occasionally | Often | Continuously |
| … the necessity to clear your throat? | No | Yes: |  | Seldomly | Occasionally | Often | Continuously |
| … the urge to cough? | No | Yes: |  | Seldomly | Occasionally | Often | Continuously |
| … problems with coughing up? | No | Yes: |  | Seldomly | Occasionally | Often | Continuously |

**Supplementary Table 2: Stop criteria**

| *Subjective parameters* | *Threshold* |
| --- | --- |
| Dizziness, discomfort and stress | Scores higher than 7 on a 10-point Likert scale (higher is worse) |
| *Vital parameters* | |
| Systolic or diastolic blood pressure drop | > 20 mmHg |
| Heartrate | >140/min |
| Respiratory rate | >25/min |
| Oxygen saturation | <80% |
| *Arterial blood gas (during screening day)* | |
| pO2 | <40 mmHg (5.33 kPa) |
| pCO2 | <25 mmHg (3.33 kPa) |
| pH | >7.55 |
| *Other* | |
| Occurrence of any other serious adverse event, or the necessity to intervene for the participant’s wellbeing. |  |

**Supplementary Table 3: Feasibility questionnaire, scored on 10-point Likert scale**

| Demand   - I would like to undergo this intervention more frequent if I am at home and it’s safe |
| --- |
| Acceptability   - Intervention was comfortable - Breathing through the mask was difficult. - Breathing through the mask was frightening. - The mask gave me claustrophobic feelings. - The intervention was distress to undergo. - The intervention took too long. - The intervention was exhausting - The intervention was painful. - The intervention made me short of breath. - The intervention gave me headaches. - The intervention made me nauseous. - I am able to undergo this intervention more often - I would recommend this therapy to others with Parkinson’s disease |
| Implementation   - I will be able to complete the complete treatment protocol of 10 treatments. |
| Practicality   - I would feel comfortable to undergo this intervention at home under direct supervision - I would only want to undergo this intervention at home when a hospital expert is present. |
| Efficacy   - I think this therapy is conceivable as Parkinson’s disease treatment. |

**Supplementary Figure 1: Summary of outcome measurements**


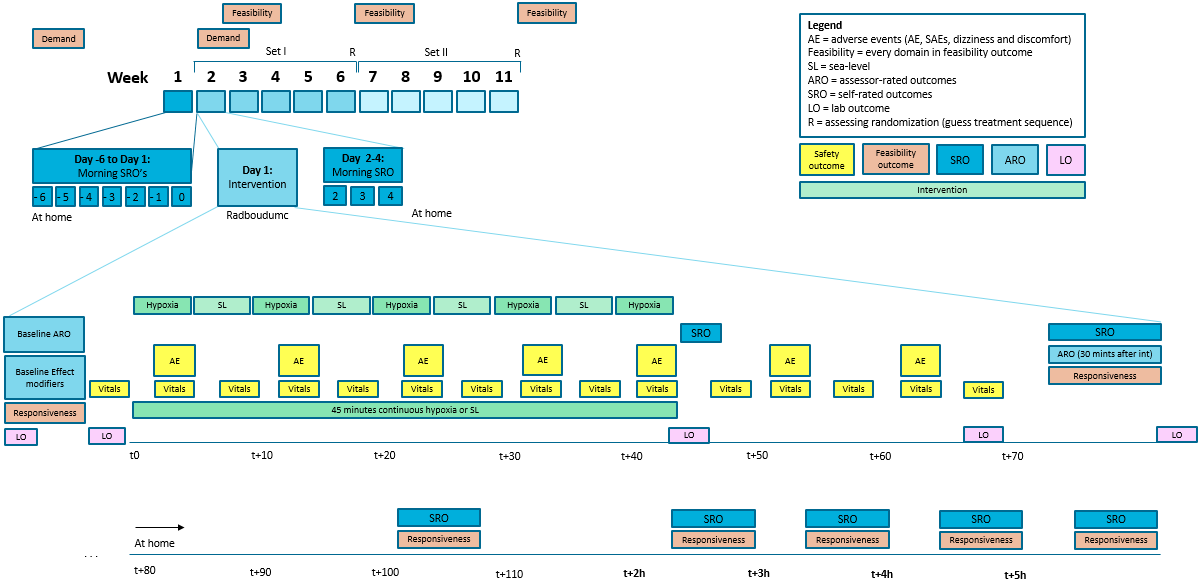

Supplement: Supplementary file 1 — Additional file 1: Supplementary Table 1. Orientating questionnaire for respiratory problems in PD [Van de Wetering et al., submitted]. Supplementary Table 2. Stop criteria. Supplementary Table 3. Feasibility questionnaire, scored on 10-point Likert scale. Supplementary Figure 1. Summary of outcome measurements [file 12883_2022_2770_MOESM1_ESM.docx]
